# Core–Shell Microfiber Encapsulation Enables Glycerol-Free Cryopreservation of RBCs with High Hematocrit

**DOI:** 10.1007/s40820-023-01213-3

**Published:** 2023-11-06

**Authors:** Xianhui Qin, Zhongrong Chen, Lingxiao Shen, Huilan Liu, Xilin Ouyang, Gang Zhao

**Affiliations:** 1 Department of Electronic Engineering and Information Science, University of Science and Technology of China, Hefei, 230027 People’s Republic of China; 2 School of Biomedical Engineering, Anhui Medical University, Hefei, 230022 People’s Republic of China; 3 Department of Blood Transfusion, The First Affiliated Hospital of USTC, Division of Life Sciences and Medicine, University of Science and Technology of China, Hefei, 230001 People’s Republic of China; 4 The Fourth Medical Center, Chinese PLA General Hospital, Beijing, 100089 People’s Republic of China

**Keywords:** Cryopreservation, Human red blood cells, Core–shell microfibers, Trehalose, Hematocrit

## Abstract

Cryopreservation of red blood cells (RBCs) provides great potential benefits for providing transfusion timely in emergencies. High concentrations of glycerol (20% or 40%) are used for RBC cryopreservation in current clinical practice, which results in cytotoxicity and osmotic injuries that must be carefully controlled. However, existing studies on the low-glycerol cryopreservation of RBCs still suffer from the bottleneck of low hematocrit levels, which require relatively large storage space and an extra concentration process before transfusion, making it inconvenient (time-consuming, and also may cause injury and sample lose) for clinical applications. To this end, we develop a novel method for the glycerol-free cryopreservation of human RBCs with a high final hematocrit by using trehalose as the sole cryoprotectant to dehydrate RBCs and using core–shell alginate hydrogel microfibers to enhance heat transfer during cryopreservation. Different from previous studies, we achieve the cryopreservation of human RBCs at high hematocrit (> 40%) with high recovery (up to 95%). Additionally, the washed RBCs post-cryopreserved are proved to maintain their morphology, mechanics, and functional properties. This may provide a nontoxic, high-efficiency, and glycerol-free approach for RBC cryopreservation, along with potential clinical transfusion benefits.

**Supplementary Information:**

The online version contains supplementary material available at 10.1007/s40820-023-01213-3.

## Highlights

- We developed a novel method for high-efficiency, nontoxic, glycerol-free, scalable cryopreservation of human red blood cells with a high hematocrit (> 40%).
- This method offered the potential for ultrafast and convenient applications by eliminating the need for permeable cryoprotectants, simplifying the washing steps, and reducing the washing time.
- This method achieved a high RBC recovery of up to 95%, and the RBCs post-cryopreserved maintained their morphology, mechanics, and functional properties.

**Supplementary Information:**

The online version contains supplementary material available at 10.1007/s40820-023-01213-3.

## Graphical abstract

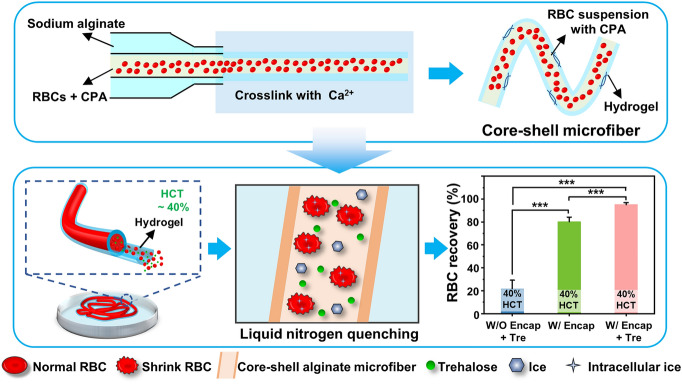

## Introduction

Red blood cells (RBCs) transfusion is a crucial component of modern medicine, providing life-saving treatment for patients suffering from acute blood loss due to traumatic or surgical hemorrhage, reduced RBC production in the bone marrow (leukemia), or decreased RBC survival in hemolytic anemias [1]. Hypothermic storage of RBCs enables blood banks to ensure a readily available and safe blood supply. However, blood can only be hypothermic preserved for up to 42 days, which can cause shortages of blood if there is a huge demand for emergency blood transfusions in emergency conditions such as natural disasters or wars [2–4].

Cryopreservation is the only reliable method for long-term storage of living cells, as cellular activities can be stopped for decades at cryogenic temperatures of − 196 °C [5]. Cryopreserved blood is just as effective as fresh blood [6], and as a result of cryopreservation, RBCs can be stored indefinitely [7]. This enables the use of blood in remote areas [8] and mitigated blood supply restrictions due to disasters or when stocks are limited [3, 9]. For the cryopreservation of RBCs, two methods are generally employed: high glycerol slow cooling technique and low-glycerol rapid cooling technique [10, 11]. Currently, the former is commonly used in the USA and Europe with ~ 40% glycerol at − 80 °C, and the latter is used in Europe with ~ 20% glycerol at − 196 °C [12]. However, severe hemolysis always occurs since glycerol with a high concentration can lead to serious osmotic imbalances during the adding and removing processes [13]. Hence, RBCs must undergo a tedious deglycerolization process before transfusion, which can minimize the amount of glycerol in RBCs and prevent adverse reactions due to excessive changes in osmotic pressure [14]. Unfortunately, this process can be time- and energy-consuming, potentially delaying the treatment in emergency cases and limiting the use of cryopreserved RBCs to rare blood phenotypes, autologous RBC storage, and military applications for blood storage [15]. Although an automated ACP 215 machine is currently available to remove glycerol, there are still problems such as the long operation time (1–2 h) and severe hemolysis of red blood cells (~ 15%) [16, 17].

Therefore, in order to minimize and eventually eliminate glycerol for RBC cryopreservation, new biocompatible cryoprotective agents (CPAs) have been investigated, including sugars, amino acids, small molecule phenolic-glycosides, osmotic balance agents, ice inhibition materials, and polymer mimics of antifreeze proteins [18–34]. Among them, trehalose has become a particularly attractive CPA as it has shown outstanding results in preventing freezing damage by stabilizing proteins and membranes, enhancing the vitrification of the solutions, as well as suppressing ice formation [35]. Subsequently, many methods based on trehalose have been developed, including predehydration [36], the intracellular delivery of trehalose [29, 30, 37–41], and so on. However, these methods are still challenging due to the too complex operation process (for example, the incubation time was up to more than 20 h), severe hemolysis during incubation, not high enough RBC recovery (for example, less than 80%), low efficiency of intracellular trehalose delivery and questionable FDA approval [20, 29, 30, 37–40], thus impeding promoting in the clinic. Moreover, although existing studies have achieved the low-glycerol cryopreservation of RBCs [42, 43], the major obstacle remains the low hematocrit levels (~ 10%) that necessitate significant storage space and additional concentration process before transfusion, hindering the practical application. Furthermore, these methods cannot further increase the hematocrit of cryopreserved RBCs, otherwise the recovery will drop drastically [29, 42, 44]. Recently, the use of alginate hydrogels to encapsulate various cells for cryopreservation has attracted wide attention [45–52], for the reason that alginate hydrogel can facilitate the vitrification of water encapsulated in small hydrogel microcapsules at low concentrations of CPAs, and suppress the formation of fatal ice crystals in the warming process [53, 54]. Our previous study has proved that hydrogel encapsulation could enhance the RBC cryopreservation with low concentrations of glycerol, although the recovery was not high (< 80%) [55]. As far as we know, core–shell structured hydrogel encapsulation has not been explored for the cryopreservation of human RBCs.

Here, we reported a novel method to successfully achieve the glycerol-free cryopreservation of human RBCs at a high final hematocrit (> 40%) by using a low concentration of trehalose as the sole cryoprotectant and core–shell alginate hydrogel microencapsulation. We found that a high RBC recovery (95.20 ± 1.68%) could be achieved after cryopreservation. What is more, the RBCs encapsulated in the hydrogel microfibers maintained integral morphology and functional properties allowing for the further clinical transfusion application. This method will not only significantly improve the efficiency of human RBC cryopreservation and reduce the storage space, which is due to the larger number of RBCs per unit volume (owing to the high hematocrit), but also simplify the process prior to clinical infusion of frozen RBCs, which will bring great convenience to clinical transfusion medicine.

## Experimental Section

### Materials and Reagents

Sodium alginate (SA) was purchased from Aladdin Industrial Corporation (Shanghai, China). Calcium chloride (CaCl_2_) was purchased from Hushi Laboratorial Equipment Corporation (Shanghai, China). Sodium citrate (SC) was purchased from Biosharp (Hefei, Anhui, China). Trehalose (Tre) was purchased from Sinozyme Biotechnology (Nanning, Guangxi, China). Sodium chloride (NaCl), glycerol (Gly), and mannitol were purchased from Sangon Biotech (Shanghai, China). All the solutions were made with deionized water. There were two kinds of cryoprotective solutions in this work. One was Tre solution used at concentrations of 0.2, 0.4, 0.6, 0.8, 1, and 1.2 M. In order to compare with the traditional low-glycerol rapid cooling method, the 35% (w/v) glycerol solution [12] was used as a control experimental group. The 35% glycerol solution consists of 3.5 g Gly, 0.3 g mannitol, and 0.065 g NaCl per 10 mL [12]. Moreover, sodium alginate was used at 0.5%, 1%, 1.5%, 2%, 2.5%, and 3% (w/v). Sodium citrate was used at 0.1, 0.2, 0.3, and 0.4 M. CaCl_2_ was used at 0.15 M with saccharides of a corresponding concentration.

### Preparation of RBCs

The human RBC suspensions used in this work were obtained from Hefei Blood Center (Anhui, China). The ethics approval was obtained from the Medical Research Ethics Committee of The First Affiliated Hospital of USTC (ChiCTR1900021038). What is more, the red blood cell suspensions were donated by different healthy volunteers. The RBC suspension was refrigerated at 4 °C for less 2 weeks. Prior to commencing the experiments, the RBC suspension underwent three rounds of washing with saline solution through centrifugation (using the Centrifuge 5702 R, Eppendorf, US) at 2500×*g* for 5 min. The supernatant was discarded at each washing. Finally, packed RBCs with a hematocrit (HCT) level of about 80% were obtained. The HCT level was measured through the capillary blood centrifugation (TG12M, Xiangyi, China).

### Adding CPA

For the 17.5% Gly groups, the packed RBCs were mixed with above-mentioned glycerol solution in a 2 mL cryovial (Cryogenic Vials, Biosharp, China). For the microfiber groups, the packed RBCs were mixed with Tre solutions in a 5 mL EP tube (BS-15-M, Biosharp, China) at an appropriate volume rate to give an HCT of 20%, 40%, and 60%. The working concentrations of the Tre solutions were 0.17, 0.33, 0.5, 0.67, 0.83, and 1.0 M. And the working concentration of Gly solution was 17.5% (w/v). Thereafter, all samples were left at room temperature for 5 min to achieve equilibrium. In addition, a fresh group that had added 0.9% NaCl only was prepared.

### Fabrication of the RBC-Laden Core–shell Structured Microfibers

The sodium alginate solutions with a concentration of 2% (w/v) were prepared as shell solutions. And the RBC suspensions with Tre were prepared as core solutions. The experimental platform for the fabrication of microfibers was built by a coaxial needle and two syringe pumps (NPZ-010, Nano Apparatus, China). Then, the RBC-laden core–shell structured microfiber was fabricated from core solutions and shell solutions with 0.15 M CaCl_2_. The size of the microfibers was controlled by adjusting the flow rates of core solutions and shell solutions. Finally, the RBC-laden microfibers were collected in a Petri dish.

### Freezing and Thawing

For the 17.5% Gly group, the cryovial was immersed vertically into the liquid nitrogen (LN_2_) for freezing. Then, the cryovial was removed from the liquid nitrogen after at least 30 min and thawed in a 40 °C water bath immediately. For the microfiber group, the surface-dried RBC-laden microfibers in the Petri dish were directly immersed in LN_2_ for cryopreservation. Then, the frozen microfibers laden with red blood cells were thawed by the pre-warmed (to 40 °C) sodium citrate solutions held in the 50 mL centrifuge tubes. The hydrogel microfibers were shaken gently and dissolved within half a minute. Additionally, the fresh group did not undergo the freezing process.

### Thermal History Measurements During Freezing and Thawing

During the cooling and warming processes, the data acquisition system (Agilent, USA) with the 34970A and 34901A module was used to detect the transient temperatures of the samples. In the operation, the tip of the T-type thermocouple was put into the microfiber or cryovial, and then temperatures were detected and recorded during freezing in LN_2_ and thawing in 40 °C water bath. And the cooling and warming rates were calculated by the software Origin2020 from the temperature curves.

### Determination of RBC Recovery

RBC recovery was determined by evaluating the amount of hemoglobin in the supernatants of the samples. The thawed RBC suspensions were centrifuged at 2500×*g* for 5 min to collect the supernatants. Then, the optical density (OD) of the supernatants at 415 nm was measured by the microplate photometer (Thermo Multiskan FC, Thermo Scientific, US). The hemolysis was calculated according to the Eq. (1). And the RBC recovery was calculated according to the Eq. (2) [56]:

1 $${\text{Hemolysis}}\; (\% ) = \left( {\frac{{A - A_{0} }}{{A_{1} - A_{0} }}} \right)*100 \%$$

2 $${\text{RBC}}\;{\text{Recovery}} \;(\% ) = 100 \% - \left( {\frac{{A - A_{0} }}{{A_{1} - A_{0} }}} \right)*100 \%$$

where *A* is the optical density for the sample supernatant, *A*_0_ is the optical density of the supernatant in the positive control group, and *A*_1_ is the optical density of the supernatant in the negative control group.

### Washing Process of Thawed RBCs

After thawing, all samples underwent a 5-min centrifugation (2500×*g*) and the supernatants were removed. Furthermore, the packed RBCs were pipetted into a 15 mL centrifuge tube. Then, for the microfiber group, saline solution was used to wash RBCs in three washing steps. In the case of the 17.5% Gly groups, adopting a standard washing process, the sodium chloride solution with a concentration of 3.5% (w/v) was utilized in the first washing step and the sodium chloride solution with a concentration of 0.9% (w/v) was used in the following two washing steps. What is more, the optical density of supernatants was measured after every washing step to evaluate hemolysis. Also, using the freezing point osmometer (Osmometer 3250, Advanced Instruments, USA), the osmotic pressure of the supernatants at every washing step was measured. After three washing steps, the RBCs were collected for the following evaluation.

### Evaluations of RBCs After Cryopreservation

#### Osmotic Fragility

Utilizing stepwise dilution of saline in the range of 0–0.9% in 0.09% increments, the osmotic fragility of erythrocytes after cryopreservation was determined. Specifically, the packed RBCs were collected first after washing. 10 μL of packed RBCs were mixed with 990 μL of the above dilutions. And then, these RBC suspensions were left at room temperature and equilibrated for 2 h. After centrifugation, the absorbance of supernatants was tested. In addition, the concentration of sodium chloride solution at 50% hemolysis rate was calculated and this was taken as the RBC osmotic fragility index.

#### Morphological Evaluation

##### Scanning Electron Microscope (SEM)

After cryopreservation and washing processes, the packed RBCs were fixed for 30 min using the 3% glutaraldehyde, and washed three times in phosphate buffer solution to completely remove the glutaraldehyde. The cells were then sequentially dehydrated once in 30% (v/v), 50% (v/v), 70% (v/v), 80% (v/v), and 95% (v/v) alcohol, and three times in 100% (v/v) alcohol for 10 min. The RBCs were vacuum dried, gold-coated, and then imaged using the SEM (JSM-6390, JEOL, Japan) at 2 kV. The magnifications were 1000 and 5000.

##### Flow Cytometry

The approximate 5 μL packed RBCs were directly resuspended in the 500 μL of PBS. Before flow cytometry, the RBC suspension was mixed gently. Then, the data analysis about washed RBCs from the three groups was performed using flow cytometry (CytoFLEX, Beckman, USA).

#### ATP

The ATP level of the RBCs after freezing, thawing, and washing was tested by the ATP Assay Kit (S0026, Beyotime, China). The procedure was completed in accordance with instructions.

#### ROS and NO

The intracellular ROS levels and intracellular NO levels were measured by the flow cytometry (CytoFLEX, Beckman, USA) using ROS Assay Kit (S0033S, Beyotime, China) and NO Assay Kit (S0021S, Beyotime, China), respectively. The negative control groups (unstained fresh red blood cells and unstained washed red blood cells after cryopreservation) were not labeled and served as the baseline for the autofluorescence detection.

### Ice Inhibition Experiment

The assessment of the ice inhibition activity was performed by a cryomicroscopy, which was composed of a microscope (BX-51, Olympus, Tokyo, Japan) and an embedded temperature-controlled cryostorage (INSTEC-HCS302GXY, Colorado, USA). The IRI activity was evaluated by “splat cooling method.” Specifically, the dispersion solution with a volume of 10 µL was dropped onto a precooled (to − 80 °C) silicon slide (which was put on an aluminum block) from a height of two meters. A solid ice film was then formed on the silicon slide. Subsequently, the frozen ice film was transferred to a cryomicroscope, annealed and lasted for 50 min. The ice crystal pictures were captured by a digital camera.

### Statistical Analysis

All results are given as the mean ± standard deviation of at least three independent experiments. Statistical significance among three groups or more was assessed using one-way or two-way ANOVA with a Bonferroni post hoc correction depending on one or two independent variables factors. A *p*-value less than 0.05 was considered statistically significant. *p < 0.05, **p < 0.01, ***p < 0.001, NS indicates that there was no significant difference between them.

## Results and Discussion

### Fabrication of RBC-laden Core–shell Structured Microfibers

**Fig. 1:**
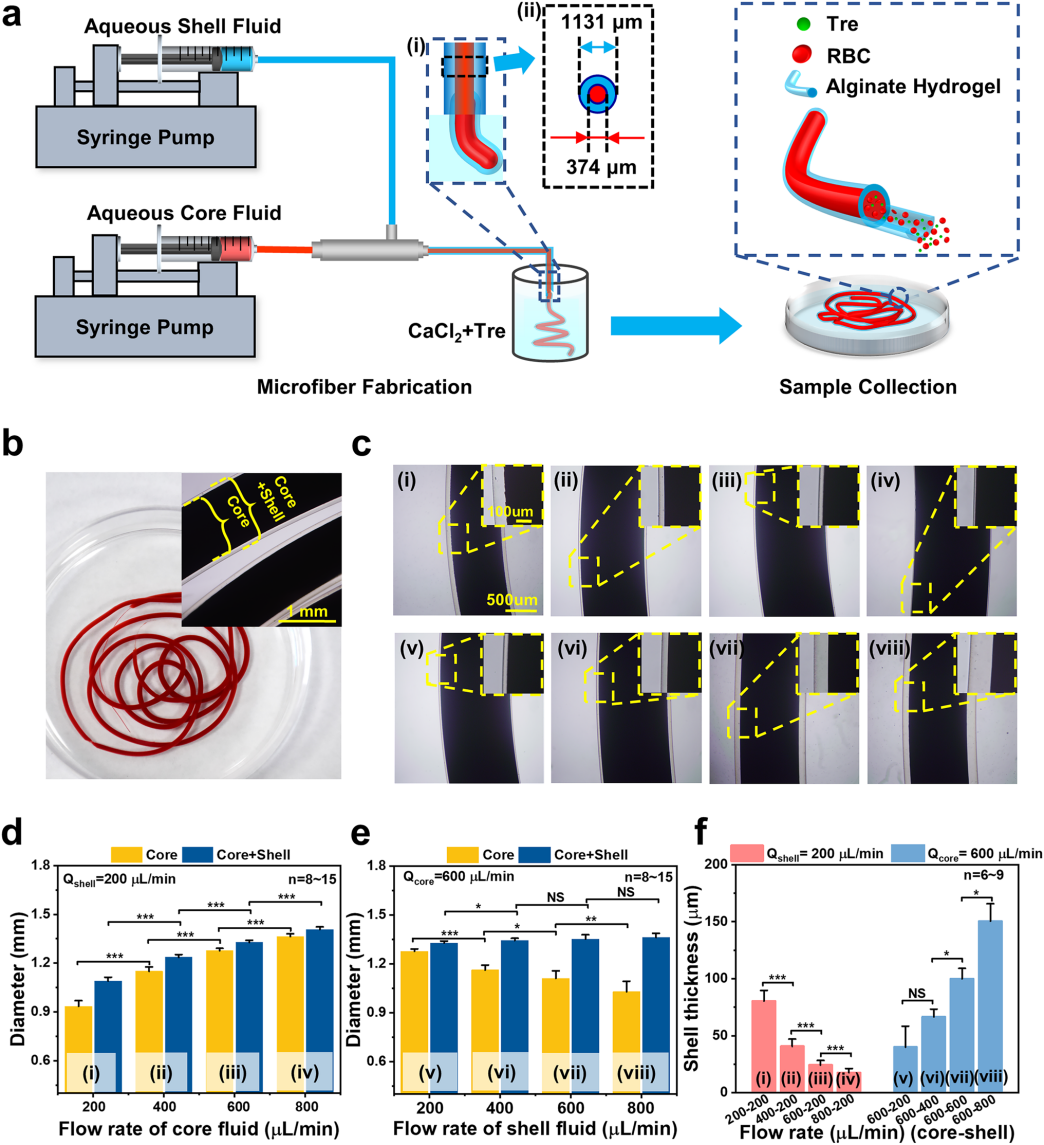
Illustration of the production process of the RBC-laden core–shell structured microfiber, morphology, and size of the hydrogel microfibers at different flow rates. **a** Overview of the microfluidic system to generate microfibers and schematic diagram of microfibers: RBC-laden alginate hydrogel microfibers with a core–shell double-layer structure. Aqueous Core Fluid: RBC suspension with trehalose. Aqueous Shell Fluid: 1%, 1.5%, 2%, 2.5%, 3% (w/v) sodium alginate solutions. RBC, red blood cell. Tre, trehalose. **b** Photographs of the core–shell structured alginate hydrogel microfibers, which were suspended in CaCl_2_ solution in a dish, and microscopic images of the microfibers. **c** (i–iv) and **d** Microscopic images and the diameters of the microfibers under different conditions: *Q*_core_ = 200, 400, 600, and 800 μL min^−1^ while *Q*_shell_ = 200 μL min^−1^. **c** (v–viii) and **e** Microscopic images and the diameters of the microfibers under different conditions: *Q*_shell_ = 200, 400, 600, and 800 μL min^−1^ while *Q*_core_ = 600 μL min^−1^. **f** The relationship between shell thickness and different flow rate combinations. *Q*_shell_: flow rates of shell fluids. *Q*_core_: flow rates of core fluids

Figure 1a illustrates the experimental devices used to generate the RBC-laden core–shell microfibers. The coaxial needle has two inlets for injecting RBC suspension with Trehalose (Tre) and aqueous sodium alginate (SA) solution, respectively. It should be noted that the coaxial needle was immersed in the calcium chloride solutions with a concentration of 0.15 M for gelling into hydrogels. At the exit of the coaxial needle, a coaxial flow of the inner fluid (RBC suspensions with Tre) and outer fluid (sodium alginate solutions at a concentration of 2% (w/v)) was generated and the gelation occurred simultaneously, due to the fact that SA can be cross-linked with calcium ions to form calcium alginate hydrogels. Thus, the solidified core–shell alginate hydrogel microfibers were continuously generated and collected in a beaker. Afterward, microfibers were collected for cryopreservation and recovery studies (Fig. 1a).

The core–shell microfibers achieved are of uniform diameter and shell thickness, as shown in Fig. 1b. These core–shell structured microfibers had a size of approximately 1–1.4 mm. By changing the flow rates of inner fluids (*Q*_core_) or outer fluids (*Q*_shell_) (as shown in Fig. 1c–f), it is easy to modulate the (inner or outer) diameters of microfibers, as well as the thickness of microfiber shells. When *Q*_shell_ was kept constant (at 200 µL min^−1^), both the inner and outer diameters of microfibers increased with increasing *Q*_core_, but the shell thickness decreased (Fig. 1d, f). Additionally, when *Q*_core_ was kept constant at 600 L min^−1^, an increase in *Q*_shell_ caused the core diameters to decrease, while the outer diameters and shell thickness increased (Fig. 1e, f). These are confirmed by the bright-field images of the generated microfibers shown in Fig. 1c. Eventually, a core–shell flow rate of 600–200 µL min^−1^ was chosen to generate microfibers for the following experiments, of which the shell thickness was 24.28 ± 4.03 μm, due to the purpose of damage-free encapsulation and the fact that the alginate hydrogel shell has a physical protective effect on RBCs against external environmental damage caused by shear stress during their production [57].

### Cryopreservation of RBC-Laden Core–Shell Structured Microfibers

**Fig. 2:**
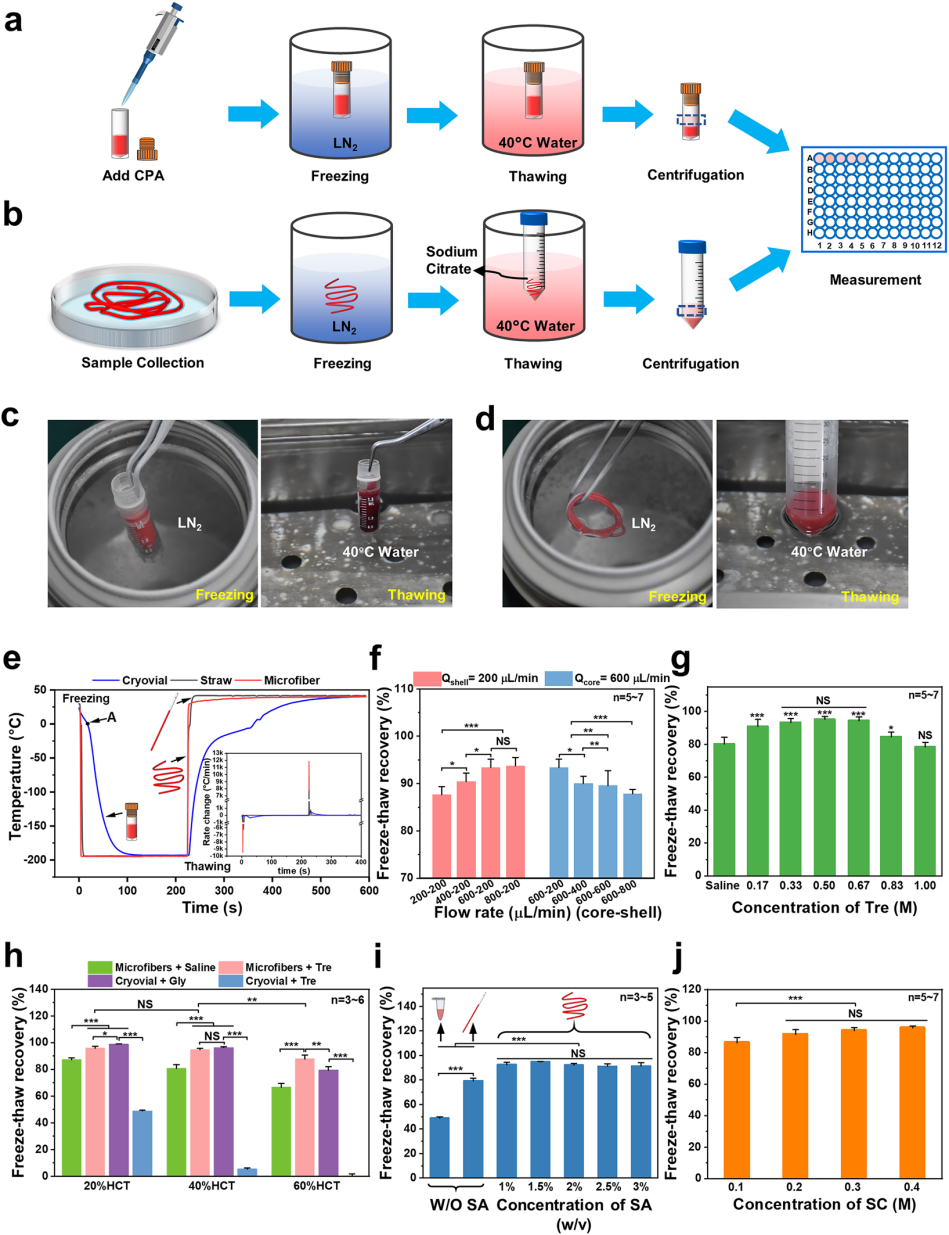
Illustration of two RBC cryopreservation processes. **a** Low-glycerol rapid cooling technology. **b** Cryopreservation of RBCs encapsulated in core–shell structured microfibers. **c** Photographs of the freezing and thawing in the low-glycerol rapid cooling method. **d** Photographs of the freezing and thawing in the cryopreservation of RBC-laden microfibers. RBC recovery under different parameters: **e** Heat transfer analysis during freezing and thawing. **f** Freeze–thaw recoveries under different flow rate combinations. **g** Freeze–thaw recoveries with Trehalose solutions at different concentrations. **h** Freeze–thaw recoveries with different groups at different hematocrits. **i** Freeze–thaw recoveries with sodium alginate solutions at different concentrations. **j** Freeze–thaw recoveries with sodium citrate solutions at different concentrations. Tre: trehalose. SA: sodium alginate. SC: sodium citrate

Both the freezing and thawing processes of 17.5% Gly group and RBC-laden microfiber group are given in Fig. 2a–d and Movies S1–S4. To analyze the heat transfer in the processes of freezing and thawing, we detected the thermal history of the 17.5% Gly group in a cryovial and the microfiber group, using a thermocouple. In general, the large temperature difference between liquid nitrogen and the sample can result in an insulating vapor film. And it may prevent the heat transfer at the border while immersing the cryovial into liquid nitrogen. This is named the “Leidenfrost phenomenon” [58]. Therefore, as shown in Fig. 2e, we could observe a turning point (point A of the blue line) during the cooling in the 17.5% Gly group with a cryovial. Before point A, the heat transfer coefficient was low. And then after point A, there was a rapid drop in the temperature profile, which indicated the vapor film disappeared. In contrast, there was no visible turning point in the microfiber group during the cooling process (as shown in the red line of Fig. 2e), which indicated that the vapor film was greatly weakened. Furthermore, the rates of freezing and thawing of the microfiber group (red line) were distinctly faster than the 17.5% Gly group in cryovial (blue line), probably for the reason that the shell thickness of microfiber (about 24.28 ± 4.03 μm) was thinner than the wall thickness of cryovial (~ 1 mm). Besides, porous alginate hydrogel may improve the heat transfer on the boundary during freezing, since previous studies have found that when porous media existed, water vapor fluxes could be “enhanced” in relation to fluxes predicted by Fick’s law [59–61], which is comparable to the vapor of liquid nitrogen. In this work, the cooling rate of the microfiber group was over 10, 000 °C min^−1^, while that of the 17.5% Gly group was about 500 °C min^−1^ (Fig. 2e). Remarkably, the warming rate of the microfiber group could be above 10,000 °C min^−1^ (Fig. 2e), allowing frozen samples to pass through the dangerous temperature zone more rapidly during warming process to avoid or reduce the possible harmful effects of recrystallization.

Considering that different shell thicknesses of hydrogel microfibers may lead to different effects on heat transfer during cryopreservation, we generated RBC-laden microfibers with different flow rates (core–shell), and the thawed RBC recovery was shown in Fig. 2f. Referring to the results shown in Fig. 1f, we could find that the thinner the shell thickness of microfibers generated successfully, the higher the RBC recovery. For instance, the microfibers with a 24.28 ± 4.03 μm shell thickness generated at the core–shell flow rate of 600–200 µL min^−1^ resulted in the RBC recovery of 95.20 ± 1.68%. However, the microfibers with a 155.96 ± 23.67 μm shell thickness generated at the core–shell flow rate of 600–800 µL min^−1^ resulted in the RBC recovery of 87.85 ± 0.91%, significantly different from that of the core–shell flow rate of 600–200 µL min^−1^. Probably because a thinner shell of hydrogel may result in a faster heat transfer [62] during cryopreservation, thus having a faster warming rate across the dangerous temperature zone during the warming process when recrystallization tends to occur [36]. Therefore, the core–shell flow rate of 600–200 µL min^−1^ was used in subsequent studies, at which it was easier to achieve the damage-free encapsulation than the core–shell flow rate of 800–200 µL min^−1^ while there was no significant difference in RBC recovery of the two flow rate combinations.

As is known, trehalose is a nonreducing disaccharide of glucose, which has been proposed as an unusual CPA due to its remarkable biocompatibility in the field of cell cryopreservation [35, 63, 64]. Therefore, the effect of Tre solutions at different concentrations on the cryopreservation of RBCs encapsulated in core–shell alginate hydrogel microfibers was investigated and the result is shown in Fig. 2g, which exhibited a typical inverted U-shaped curve trend with the Tre final concentrations varying from 0.17 to 1.0 M. As we can see from the results, either too high or too low a concentration of Tre can harm the thawed RBC recovery. For example, the freeze–thaw recovery reached 93.24 ± 2.45%, 95.20 ± 1.68%, and 94.42 ± 2.20% in the 0.33, 0.5, and 0.67 M groups (of which the 0.5 M group was higher than the other two groups), respectively, which were significantly different with the group without adding Tre (80.24 ± 4.0%), while only 78.40 ± 2.76% in the 1.0 M Tre group, statistically insignificant compared with the group without Tre. This may be due to the cryoprotective effect of trehalose itself and its predehydration. Different concentrations of Tre solutions with different osmotic pressures can dehydrate RBCs to different degrees (Fig. S1a, b), and 0.5 M Tre can dehydrate RBCs to their minimal volume (Fig. S1c, d), thus reducing the probability of IIF during cooling. In contrast, 1.0 M Tre may result in excessive osmotic stress, which can cause damage to cells. These results demonstrated that trehalose can effectively improve the post-cryopreserved recovery of RBCs encapsulated in microfibers. Notably, we found that the encapsulated RBCs still have a high recovery (80.24 ± 4.0%) post-thawing even without adding CPAs, as shown in the column on the far left in Fig. 2g. But that’s a small wonder because the cooling rate in the core–shell microfibers can reach 10,000 ℃ min^−1^, as shown in Fig. 2e. Our results are similar to previous studies, which have already proved that as long as the cooling rate was fast enough, there was a survival rate of ~ 50% when isotonic saline was used only [65–67]. But the RBC recovery using saline only in our experiment was higher, probably because the RBCs were encapsulated in the core–shell microfibers, which kept the RBCs apart from the ice crystal that had formed outside. Eventually, we confirmed 0.5 M as the final concentration of Tre for the following experiments.

Considering that the cell concentration has an impact on the cryopreservation, and that hematocrit is another form of concentration for RBCs [68], it is essential to determine a hematocrit level suitable for the RBC cryopreservation in our work. Figure 2h shows the recoveries of RBCs treated with or without adding Tre cryopreserved according to the procedure shown in Fig. 2b, together with the non-encapsulated RBCs cryopreserved by the traditional method shown in Fig. 2a. Clearly, in the four groups, the higher the final hematocrit, the lower the RBC recovery due to the “packing effect,” which resulted from the fact that the channels formed by unfrozen liquid during freezing led to cell accumulation and the impact of mechanical stress [69]. For the microfiber group with Tre, the hematocrit level of 20% showed a high recovery of 95.89 ± 1.46%, which was not significantly different from that of the 17.5% Gly group. When the final hematocrit level was increased to about 40%, the recovery was 94.82 ± 1.01%, still as high as that of 20% HCT. And the difference in RBC recovery between the microfiber group with Tre and the 17.5% Gly group is not significant. However, once increasing the final hematocrit to 60%, the RBC recovery of the microfiber group with Tre (87.75 ± 3.04%) was significantly higher than the 17.5% Gly group (79.42 ± 2.65%), although both of them dropped. In brief, the final hematocrit of 40% was chosen, since the cryopreservation of RBC suspension of a high hematocrit level will be more efficient than that of a low hematocrit level in clinical applications, although it has been difficult when the requirement of high recovery was met.

Moreover, when we cryopreserved RBCs with Tre as the sole CPA in the cryovial, the recovery decreased significantly (shown in Fig. 2h). To explain this, we first evaluated the mechanical property of the RBC microfiber through the tensile test (Fig. S2a–i) because of its elongated shape. Due to the cylindrical shape of the cryovial, which makes it difficult to create an “H” shape that meets the testing requirements, we conducted a compression test to evaluate its mechanical performance (Fig. S2a-ii). As shown in Fig. S2a, the RBC microfibers can tolerate up to 120% strain at room temperature before rupture, which means good toughness. Cryovials, however, can only tolerate less than 80% of the strain in the compression test before rupture occurs, indicating that they are vulnerable to mechanical stress. In addition, we calculated the elastic modulus of microfibers and cryovials (shown in Fig. S2b) and found that the elastic modulus of microfibers was significantly smaller than that of cryovials, which means that the microfibers are more prone to deformation and bending when subjected to mechanical stress during cryopreservation, resulting in the release of internal stress. This may be the reason for the higher recovery in the Microfiber + Tre group compared to the Cryovial + Tre group. It should be noted that the elastic modulus of the material (polypropylene) of the cryovials used in this experiment is typically in the range of several GPa [70], much larger than that of the cryovials we tested. In addition to the difference in elastic modulus, the cooling and warming rates of the cryovials are also significantly lower than those of the microfibers (Fig. 2e). As the optimal cooling rate depends on the CPA (type and concentration) [71], the optimal cooling rates of glycerol and trehalose are different. Considering the results from the Microfiber + Tre, Cryovial + Tre, and Cryovial + Gly groups (Fig. 2h), we speculated that glycerol might have a relatively lower optimal cooling rate [72–74].

Generally, the concentrations of sodium alginate solutions can affect hydrogel microfibers’ toughness and the SA solution with a concentration over 3% (w/v) is too sticky [75]. Therefore, we investigated the effects of SA solutions at different concentrations ranging from 1 to 3% (w/v) on RBC recovery and the results are shown in Fig. 2i. It was easy to see that when Tre was used as the sole cryoprotectant, the method in which RBCs were encapsulated in core–shell hydrogel microfibers for cryopreservation reached a significantly higher recovery (up to 95.20 ± 1.68%) at all SA concentrations than the unencapsulated cryopreservation in EP tubes (48.93 ± 1.08%) and straws (79.30 ± 2.16%). The reason for these results may be as follows: First, the CPA trehalose can dehydrate the RBCs before cooling, reducing the intracellular ice crystals [36]. Second, the elastic modulus of microfibers was really small, which means that the microfibers are more prone to deformation and bending when subjected to mechanical stress during cryopreservation, resulting in the release of internal stress. Third, alginate hydrogels can suppress ice crystal growing through adhering to the surface of the initial ice crystals too [75]. Finally, during cryopreservation, the shell of the microfibers could not only facilitate the heat transfer through inhibiting the film boiling, but also protect the encapsulated cells from mechanical damage from the outside, acting as a physical barrier [49]. Therefore, ultra-rapid cooling and warming can effectively suppress the ice forming inside and outside the cell, protecting the RBCs from mechanical damage. Since the RBC recovery did not differ significantly across all groups with encapsulation, the 2% SA solution was chosen, which was not too sticky and can produce hydrogel microfibers with suitable toughness. Furthermore, to investigate whether the concentration of SC would affect the cryopreservation of RBCs during the thawing process, we detected the RBC recovery of four groups (0.1, 0.2, 0.3, and 0.4 M SC solutions, respectively), as shown in Fig. 2j. The concentration of 0.3 M was chosen because its osmotic pressure was suitable for our following washing process.

### Assessments of the Thawed RBCs After Washing

#### Washing Process of the Thawed RBCs

**Fig. 3:**
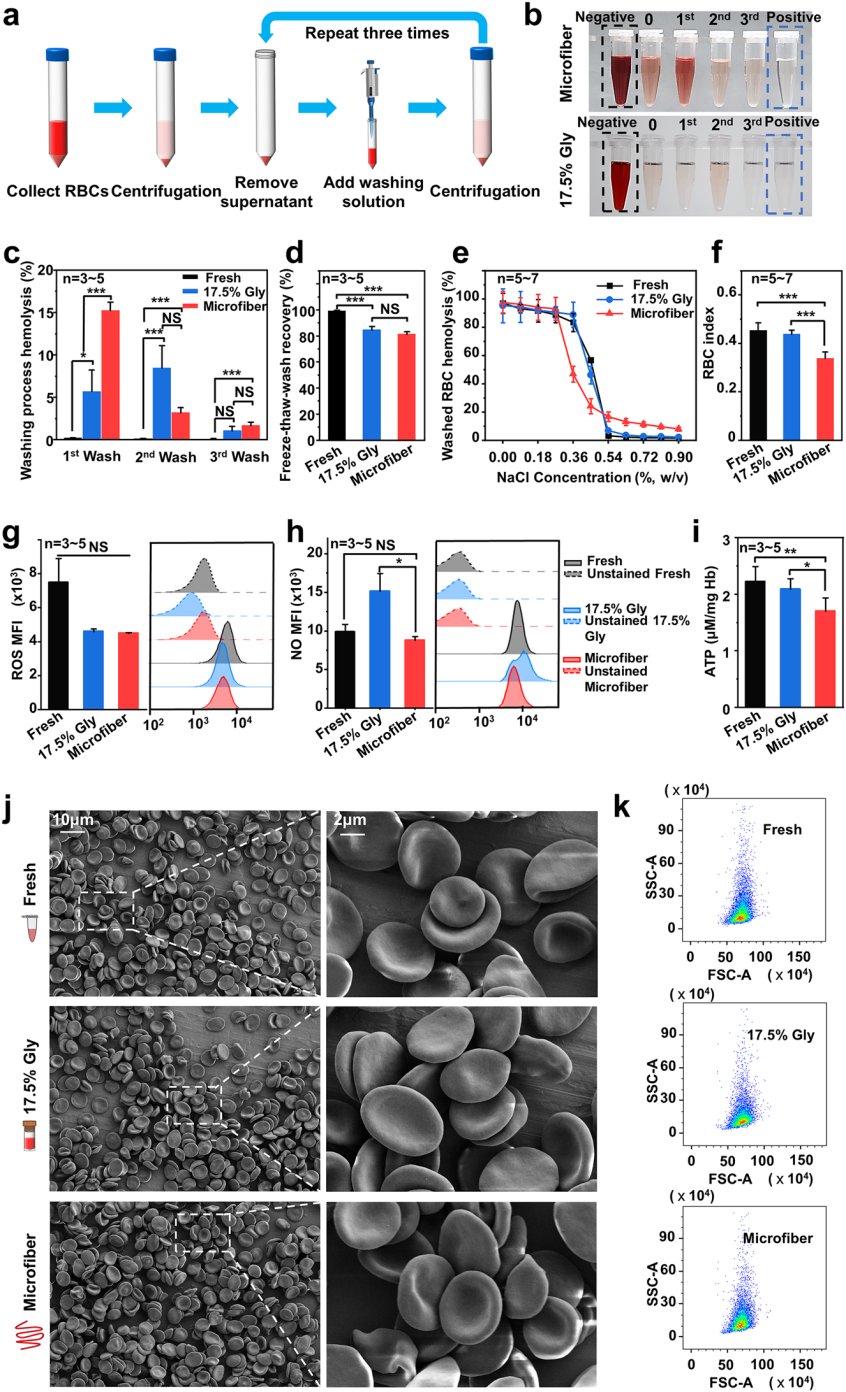
Washing process and assessments of RBC functional properties and RBC morphology after washing. **a** Diagram of the RBC washing process. **b** Photographs of the supernatants at each step during the RBC washing of the two cryopreservation methods. **c** Hemolysis at each step during washing process. **d** The freeze–thaw–wash recovery of different groups after the washing process. **e** Osmotic fragility curves of washed RBCs with NaCl solutions at different concentrations. **f** Osmotic fragility index of red blood cells after washing. Functional properties for fresh RBCs (black), washed RBCs after cryopreservation in the 17.5% Gly groups (blue), and the microfiber groups (red) were compared. Three kinds of biochemical properties of RBC were evaluated: **g** intracellular ROS level, **h** intracellular NO level, and **i** ATP levels of washed RBCs after cryopreservation. The negative controls (unstained fresh RBCs indicated by black dotted lines, unstained washed RBCs in the 17.5% Gly groups represented by the blue dotted line, and unstained washed RBCs in the microfiber groups represented by the red dotted line) were not labeled and were used as a baseline to detect the autofluorescence of cells. Assessment of RBC morphology following cryopreservation and washing process: **j** electron microscopic observations of the RBCs. **k** The scatter plots obtained by the flow cytometry

Since the highest RBC freeze–thaw recovery following cryopreservation by encapsulating red blood cells in core–shell alginate hydrogel microfibers was achieved by adjusting every parameter to the optimal (600–200 µL min^−1^ (core–shell), 0.5 M Tre, 40% Hct, 2% SA, 0.3 M SC), further assessment on the red blood cells’ quality was focused on this group. Before the quality parameters were investigated, we have to go through the washing process, as shown in Fig. 3a. During the washing process, the typical photographs of the supernatant are shown in Fig. 3b, which reflected the hemolysis of washed RBCs, and the corresponding hemolysis rate in each step of washing process together with the RBC recovery post-washed are exhibited qualitatively in Fig. 3c, d. Although the osmolality changes undergone by thawed RBCs of the microfiber groups and the 17.5% Gly groups were different (Fig. S3), there was no significant difference in the washing hemolysis between the two groups in the 2nd wash and 3rd wash processes, except for the 1st wash process. It is inspiring that the post-washed RBC recovery of the microfiber group is up to 80%, which is not significantly different from the 17.5% Gly group. It should be noted that the washing process is more simplified and time-saving than traditional cryopreservation with glycerol [10, 11], taking about 20 min (manual washing in three steps). However, the removal of glycerol, despite the use of automated equipment, still takes 0.5–2 h (30 min in three steps in the low-glycerol rapid freezing method, and 1–2 h in the high glycerol slow freezing method) and the hemolysis rate is high. This can bring great convenience to the application in emergency situations.

#### Assessment of the Biomechanical Property of RBCs Following Freezing, Thawing, and Washing

The effect of cryopreservation of RBCs encapsulated in core–shell hydrogel microfibers with the presence of sole trehalose on the biomechanical property of RBCs represented by their osmotic fragility is shown in Fig. 3e, f**.** The results show that the osmotic fragility index in the microfiber group was lower than other two groups. It means that RBCs in the microfiber group could tolerate a lower concentration of NaCl solution (with lower osmotic pressure) when the hemolysis rate was 50%. In other words, when exposed to a hypotonic environment, the microfiber group’s RBCs are less prone to hemolysis. This means that the volume regulation of washed RBCs post-cryopreservation in the microfiber group was superior to that of the other two groups, and the RBCs possessed greater stability and resilience, maintaining their integrity and avoiding damage under external pressure.

#### Assessments of the Biochemical Properties of Recovered RBCs

To assess the biochemical properties of recovered red blood cells in the microfiber group, we measured intracellular ROS level and intracellular NO level, which acts as regulators for RBC homeostasis and blood flow [76], as shown in Fig. 3g, h, respectively.

Previous studies have shown that ROS formed during the preservation could accelerate the storage injury of RBCs and notably the RBC viability post-transfused [77, 78]. Additionally, a series of cell components are targeted due to the high reactivity of ROS, consisting of nucleic acids, lipids, and proteins (such as RBC spectrin), which experience irreversible changes caused by ROS, leading to a distinct decrease in RBC deformability [79]. As an important short-lived signaling molecule, NO plays an important role in regulating local vasodilation [80]. Reduced NO levels have been reported following prolonged preservation of red blood cells, which have been connected with biochemical changes (for example, depletion of ATP) and hemolysis-related changes mainly involving the changes in morphology and deformability [81]. Our flow cytometry results proved that the RBCs recovered after cryopreservation and washing in the microfiber group maintained both the intracellular ROS and NO levels compared with fresh RBCs, although cryopreservation of the 17.5% Gly group appeared to augment the intracellular NO level of RBCs (Fig. 3g, h), which was similar to the results obtained in reference [82]. During the metabolism of RBCs, ATP is essential for maintaining electrolyte balance, as well as increasing cell deformability [83]. The ATP levels of washed RBCs from different groups are shown in Fig. 3i. Even though a decrease in the ATP level was observed in the microfiber group, a previous study has already revealed that stored RBCs could recover their ATP concentration in 37 °C human serum and this process occurred on a scale of tens of minutes [84].

#### Examination of RBC Morphology

As we know, normal red blood cells possess a characteristic biconcave and discoidal shape. And the discoid morphology of RBCs is conducive to improving the oxygen-delivering capacity thanks to the high surface area [85]. Moreover, red blood cells appear in extreme flexibility because of the biconcave shape [86], making it easy for them to pass through the microvasculature. Thus, a series of microscopic images by SEM were obtained to study the effect of cryopreservation and washing in the microfiber groups and 17.5% Gly groups on RBC morphology (Fig. 3j), including high-magnification SEMs. After freezing, thawing, and washing processes, the morphology of RBCs in both the 17.5% Gly groups and microfiber groups was indistinguishable from the morphology of fresh RBCs, indicating that the morphology of post-washed RBCs of the two groups was nearly completely intact. This is in accord with the previous observation of the biconcave morphology of human red blood cells [87]. Moreover, flow cytometry was also used to analyze the morphology of RBCs. The forward scatter and side scatter in the scatter plots, respectively, showed the size and complexity of the cell population. And no significant difference can be seen in the scatter plots among the three groups (Fig. 3k), suggesting that the RBCs were intact post-cryopreservation and washing.

In addition, we further investigated the effect of predehydration of other nonpermeable saccharides on the cryopreservation of human RBCs encapsulated in microfibers (Figs. S4–S6), including the recovery and the assessments of RBCs after cryopreservation. Specifically, the freeze–thaw recoveries of trehalose, maltose, and raffinose were not significantly different (all above 90%) and the assessment results were similar for all groups. By comparing these data, we could find that core–shell alginate hydrogel encapsulation makes it possible that membrane-impermeable saccharides do not need to exist both inside and outside the cell membrane to achieve good cryoprotective efficiency. This is probably because predehydration with saccharides can minimize the osmotic shock and IIF during cooling and most sugars possess a membrane stabilizing effect.

**Fig. 4:**
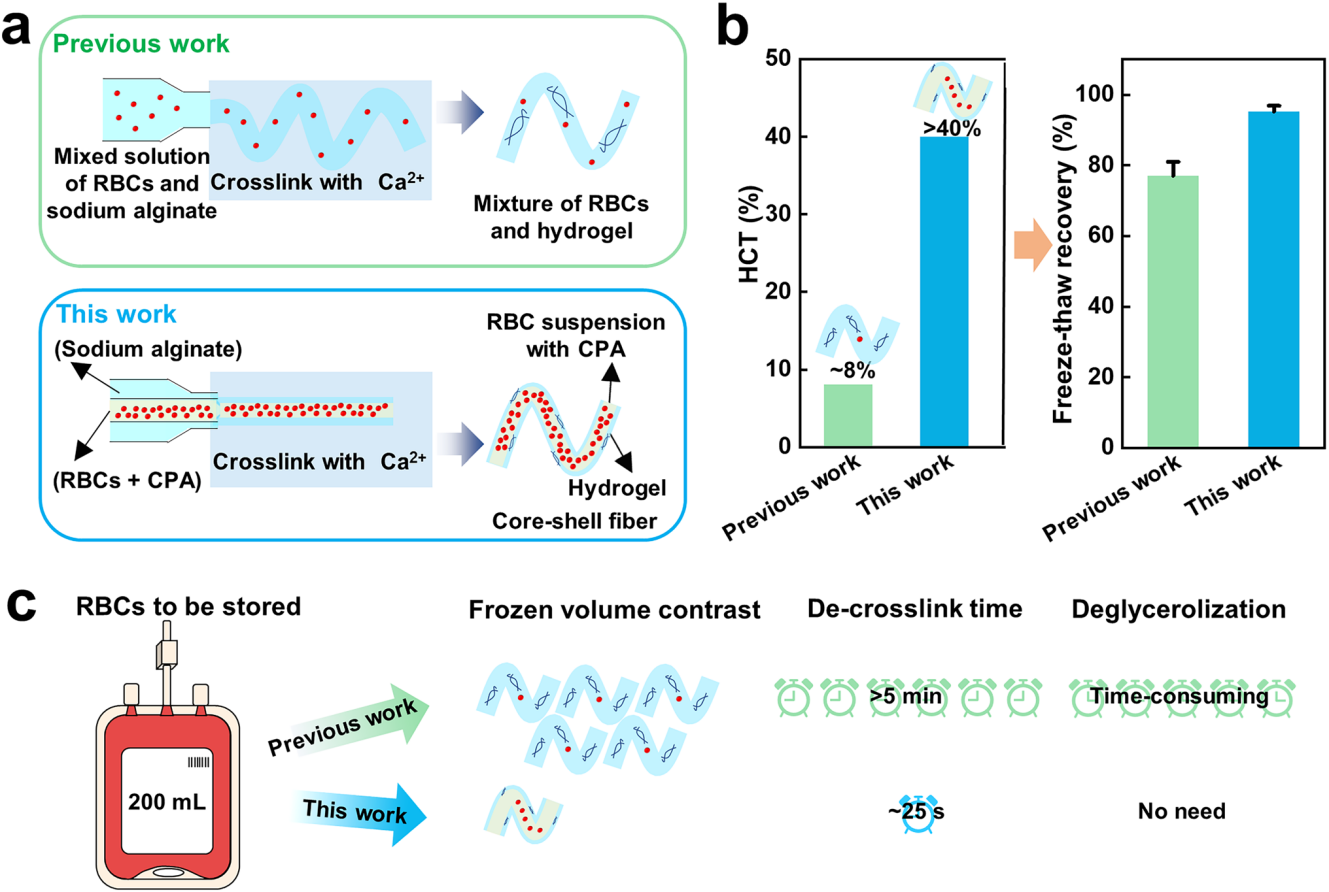
Comparison between the present and previous cryopreservation methods with RBCs encapsulated in hydrogel microfibers: **a** fabrication and structure, **b** HCT and freeze–thaw recovery,** c** process capacity

Overall, our work proved that the combination of core–shell microfiber encapsulation and trehalose predehydration had great potential for the glycerol-free RBC cryopreservation. In addition, compared with the previous work [55], our work has made great improvements, mainly in the following points: First, these are two different structures of hydrogel microfibers. The one in our work is a core–shell structured microfiber fabricated by a coaxial nozzle, while the one in previous work is a simple structured microfiber fabricated by a single nozzle** (**Fig. 4a). And the shell of the microfibers could not only facilitate the heat transfer through inhibiting the film boiling, but also protect the encapsulated cells from mechanical damage from the outside, acting as a physical barrier [49]. Secondly, our work without glycerol significantly improves (by > 15%) the freeze–thaw recovery even at a higher HCT (> 40%, at least five times as high as the reported one with glycerol)** (**Fig. 4b), which means a more efficient cryopreservation of RBCs. For example, when there is a unit of RBCs to be cryopreserved, the previous method requires more hydrogel microfibers to be fabricated, and therefore, more space, energy consumption, and de-cross-link time **(**Fig. 4c). What is more, the previous method requires a deglycerolization process (due to the use of glycerol) and an extra concentration process before clinic infusion (due to the low hematocrit), which may be inconvenient to clinical applications. It should be pointed out that HCT is one of the key parameters determining the feasible application of the RBC cryopreservation technologies.

### Possible Mechanism for the RBC Cryopreservation with the Encapsulation of Core–Shell Structured Microfibers

To further explore the effects of conventional rapid freezing method and our method, cryomicroscopy studies of the cryoprotectants used in the two cryopreservation protocols (17.5% Gly and 0.5 M Tre) were performed. As mentioned above, the cooling rate of the microfiber group was extremely rapid (up to 10,000 °C min^−1^, Fig. 2e), which cannot be achieved by the cryostage. At the same time, to prevent excessive ice crystal growth from affecting our observations, we compared the ice crystal growth of the two CPAs during the cooling process at a cooling rate of 5 °C min^−1^. The results are shown in Fig. S7, Movies S5 and S6. For better comparison, we set the time when only a single small ice nucleus with the same size was left in the field of view as 0 s, and then collected pictures of ice crystal growth over different time. The results show that the ice crystal growth rate of the trehalose group was faster than that of the glycerol group (shown in Fig. S7). This might be one of the reasons why the recovery in the Cryovial + Tre group was lower compared to Cryovial + Gly group (Fig. 2h).

**Fig. 5:**
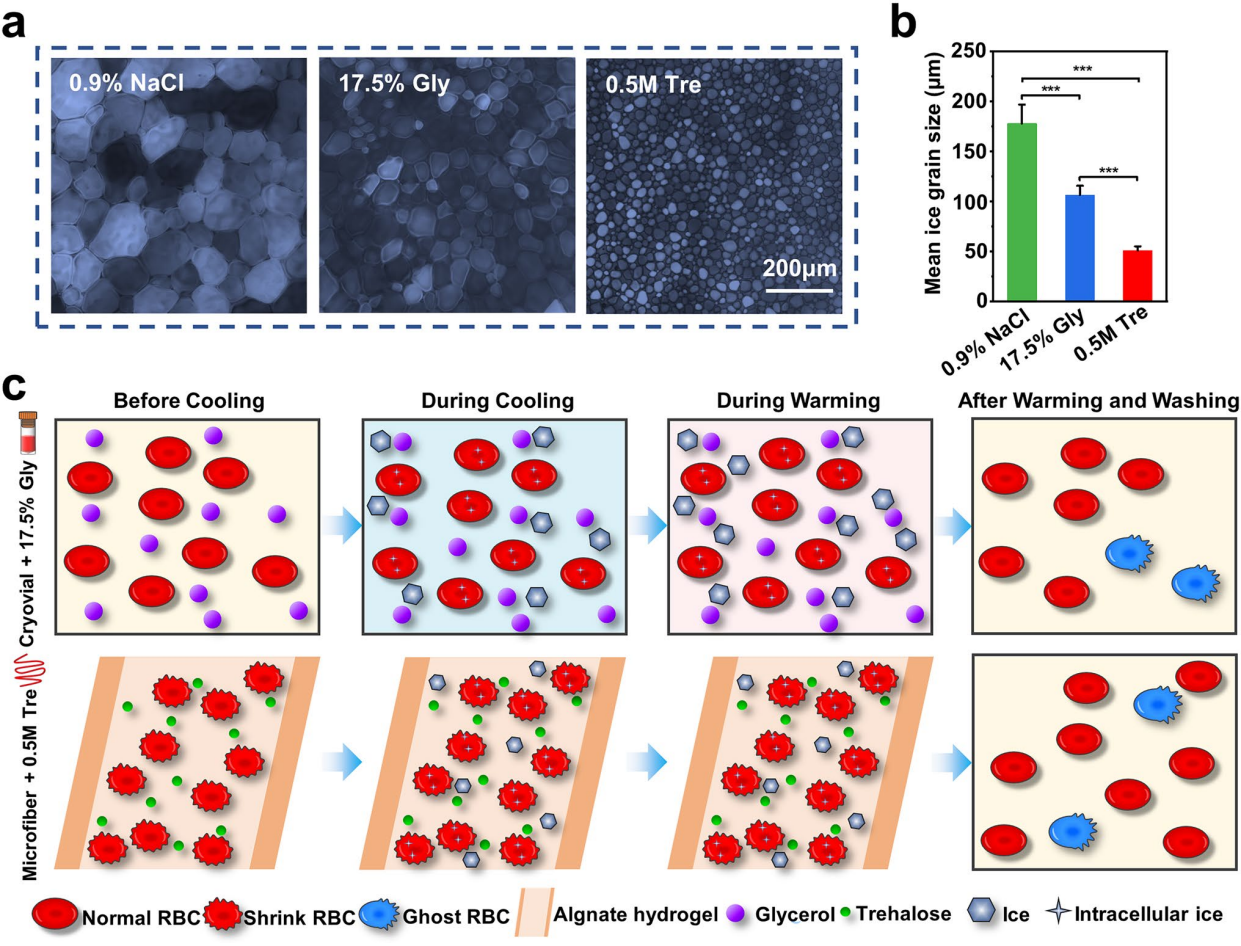
Mechanism analysis for the RBC cryopreservation. IRI activity of different CPA groups: **a** Microscopic images of ice crystals grown in the NaCl solution (0.9%), 17.5% Gly solution, and 0.5 M Tre solution. **b** Mean ice grain size of different groups. **c** Illustration of the possible mechanism during the cryopreservation

Furthermore, a “splat” assay normally used for evaluating the ice recrystallization inhibition (IRI) activities quantitatively was accomplished. In general, the smaller the size of the ice crystals means that the ability to inhibit recrystallization of the ice crystals is stronger [26, 33]. As seen in Fig. 5a, b, it was clear to see that the recrystallized ice crystals of the 0.5 M Tre group were much smaller than that of the other two groups, indicating that 0.5 M Tre had an obvious inhibitory action on ice crystal recrystallization.

Figure 5c depicts the possible mechanism for the RBC cryopreservation with core–shell microfiber encapsulation and trehalose predehydration. For the 17.5% Gly groups, glycerol could permeate into RBCs, thus protecting RBCs from damage resulting from cooling through suppressing excessive volume reduction together with fatal concentrations of electrolytes. Additionally, glycerol could reduce the chemical potential of solutions as well as the freezing point, thus preventing ice from forming [15]. As a consequence, most RBCs in the cryovial group survived. In the microfiber group, most RBCs survived post-cryopreservation, because of the following reasons: CPA trehalose dehydrated RBCs before cooling, thus reducing the formation of intracellular ice during cooling [36], and it had an obvious inhibitory action on ice crystal recrystallization (Fig. 5a, b). Then, during cryopreservation, the shell of the microfibers could not only facilitate the heat transfer through inhibiting the film boiling [46], but also protect the encapsulated cells from mechanical damage from the outside, acting as a physical barrier. The ultrafast rewarming rate (shown in Fig. 2e) allowed RBCs to pass through the dangerous temperature zone more rapidly during warming process to avoid or reduce the possible harmful effects of recrystallization. Finally, most RBCs recovered from the washing process due to the membrane stabilization effect of trehalose.

### Scalability of the Cryopreservation Method

**Fig. 6:**
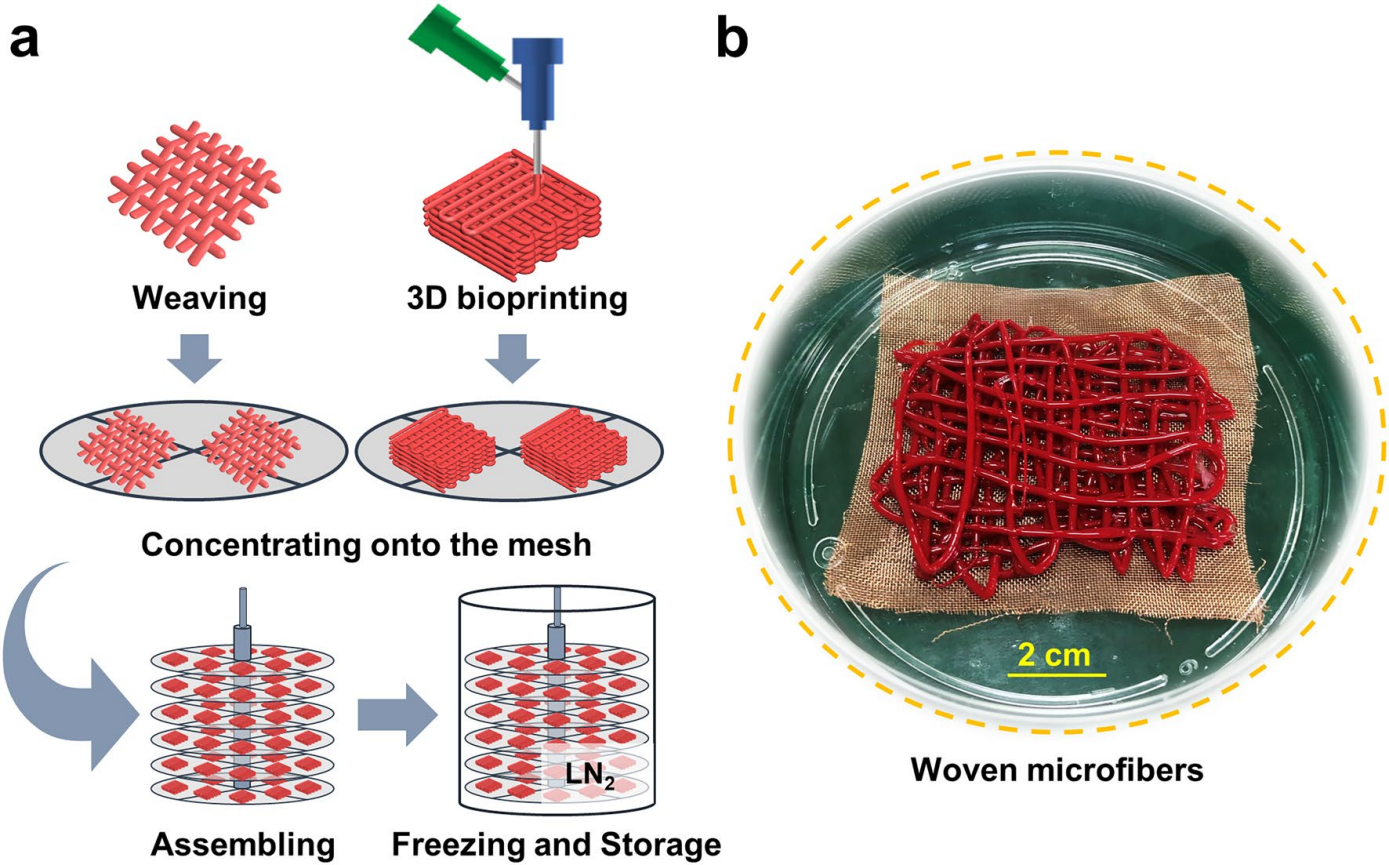
**a** Conceptual schematic of scaling-up. Core–shell RBC-laden microfibers are 3D bioprinted or woven, and concentrated onto mesh disks. Then, these mesh disks are stacked and assembled together. Finally, RBC microfibers concentrated on the assembled disks are frozen and stored in UV-sterilized LN_2_. An example of stacked mesh for freezing is displayed. **b** The picture of woven microfibers

For “ready-to-use” human red blood cells, the scalability of the cryopreservation approach is essential. Further scale-up of our method for a clinical meaningful throughput (for example, a unit of blood) can be achieved using 3D bioprinting or woven fibers, and concentrating onto cryomesh before freezing, which can be enlarged or stacked as showed in the schematic diagram of Fig. 6a. A picture of woven microfibers is displayed in Fig. 6b. Thus, when a lager mesh is plunged into liquid nitrogen rapidly and uniformly, the cooling and warming fluxes and thermal mass will remain independent of mesh size and shape, under the conditions that cooling/warming baths are sized adequately and the number of 3D bioprinting/weaving layers is not too many. In addition, cryobioprinting can provide an ideal method for scaling-up of this work, as freeform cell-loaded cryobioprinting that is used to fabricate and store “off-the-shelf” tissues has been reported [88]. Notably, the sample cooling rates during cryopreservation have an important effect on ice crystal formation and cell recovery. When a large volume of RBCs needs to be stored, a rapid cooling rate may be difficult to achieve, thus it requires far more efforts from both experimental and theoretical studies. And more work is still needed to fully investigate the functional properties of RBCs.

## Conclusion

In this work, we successfully achieved the glycerol-free cryopreservation of RBC suspension at a high hematocrit level encapsulated in core–shell alginate hydrogel microfibers with sole trehalose as CPA. Specifically, we used the core–shell structured microfibers with a rather thin shell thickness to accelerate the heat transfer and 0.5 M Tre as the sole CPA to dehydrate RBCs, enabling the successful cryopreservation of RBC suspension with a very high hematocrit level (~ 40%, two times higher at least, compared to the commonly reported cryopreservation of RBCs). With this method, the recovery of RBCs post-cryopreservation reached up to 95% and the RBCs maintained normal morphology, mechanics, and contents of the intracellular ROS and NO. Compared to traditional methods for RBC cryopreservation, our work is innovative in the following aspects: First, it offers an ideal approach for nontoxic and glycerol-free cryopreservation of human red blood cells omitting the tedious glycerolization and deglycerolization processes, while the traditional cryopreservation of RBCs requires 20% or 40% (w/v) glycerol. Second, it provides a strategy to cryopreserve the RBC suspension with a high concentration, and the microfibers provided large construct volumes enabling high-throughput capacity for rapid manufacture of mass production compared with microcapsules, thus providing a wonderful strategy for high-throughput cryopreservation of human erythrocytes. Third, the coaxial needles used in our work for the microfiber fabrication can be integrated into arrays for high throughput due to its extensibility, and further study can focus on the seamless combination of extrusion bioprinting and cryopreservation of RBCs for simultaneously fabricating and storing RBC-laden volumetric tissue constructs. What is more, our work indicates that core–shell alginate hydrogel microencapsulation makes it possible that membrane-impermeable trehalose does not need to exist both inside and outside the cell membrane to achieve good cryoprotective efficiency, which is different from existing trehalose-based RBC cryopreservation studies. Therefore, the RBC cryopreservation based on encapsulation of core–shell alginate hydrogel microfibers and predehydration of trehalose is valuable for clinical use. The principles underlying this approach may also have relevance and applicability to other cell types such as immune cells or stem cells, but further research and optimization are still needed, considering the fact that different types of cells have unique properties and requirements during cryopreservation. In a word, this work opens an attractive way to cryopreserve RBC with high hematocrits and provides the “off-the-shelf” availability in clinical transfusion.

## Supplementary Information

Below is the link to the electronic supplementary material.

Supplementary file1 (AVI 10993 kb)

Supplementary file2 (AVI 10286 kb)

Supplementary file3 (AVI 15549 kb)

Supplementary file4 (AVI 8480 kb)

Supplementary file5 (AVI 18926 kb)

Supplementary file6 (AVI 18308 kb)

Supplementary file7 (PDF 1229 kb)
