# Supplementary material for: Core–Shell Microfiber Encapsulation Enables Glycerol-Free Cryopreservation of RBCs with High Hematocrit
Source: Nanomicro Lett. 2023 Nov 6;16:3. doi: 10.1007/s40820-023-01213-3 (PMC10628128; doi:10.1007/s40820-023-01213-3)
Supplement: Supplementary file 7 — Supplementary file7 (PDF 1229 kb) [file 40820_2023_1213_MOESM7_ESM.pdf]

Supporting Information for

Core-Shell Microfiber Encapsulation Enables Glycerol-Free Cryopreservation of RBCs with High Hematocrit

Xianhui Qin[1], Zhongrong Chen[2], Lingxiao Shen[1], Huilan Liu[3, *], Xilin Ouyang[4, *] and Gang Zhao[3, *]

[1] Department of Electronic Engineering and Information Science, University of Science and Technology of China, Hefei, 230027, P. R. China

[2] School of Biomedical Engineering, Anhui Medical University, Hefei, 230022, P. R. China

[3] Department of Blood Transfusion, The First Affiliated Hospital of USTC, Division of Life Sciences and Medicine, University of Science and Technology of China, Hefei, 230001, P. R. China

[4] The Fourth Medical Center, Chinese PLA General Hospital, Beijing, 100089, P. R. China

* Corresponding authors. E-mail: zhaog@ustc.edu.cn (Gang Zhao), huilanl@ustc.edu.cn (Huilan Liu), ouy70301@sina.com (Xilin Ouyang)

S1 Supplementary Results

Saccharides are generally classified as nonpermeable cryoprotective agents, therefore can dehydrate the cells before cryopreservation for the reduced probability of intracellular ice formation (IIF), a lethal event to cells, during cooling, which has been proved in trehalose [S1]. Thus, we further tested the effects of other saccharides on the cryopreservation of human RBCs encapsulated in microfibers.

S1.1 Effect of Different Concentrations of Different Saccharide Solutions on RBC Dehydration

The hematocrit (HCT) was used to roughly assessed the degree of dehydration of RBCs by different saccharide solutions at different concentrations (as shown in **Fig. S1a**), with which the osmolality of the supernatants of RBC suspensions after incubation was also measured (**Fig. S1b**). It was easy to see that the degree of RBC dehydration increased as the osmolality of the solution increased (**Fig. S1c**). For the Tre groups, a concentration of 0.5 M could dehydrate RBCs to their minimal volume, and the hematocrit was not significantly different from 0.67 M and 0.83 M trehalose. When the concentration was increased to 1 M, an increase in RBC HCT was found in Tre group (**Fig. S1d**), which may due to the fact that 1 M Tre may result in excessive osmotic stress, and the over-dehydration of RBCs may produce rough surface (as shown in **Fig. S1a(d)**). The same trend was observed in other saccharide solutions.

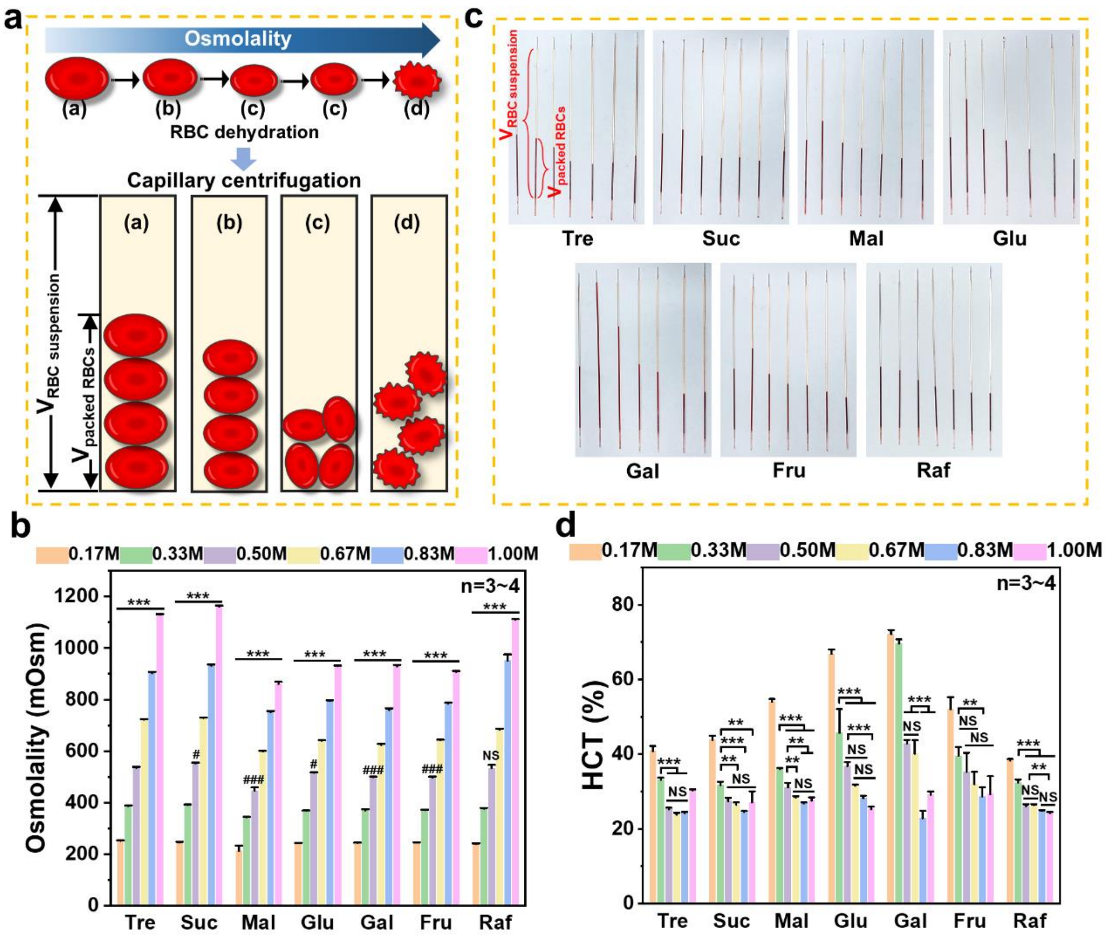

Fig. 1 a) Schematic diagram of RBCs dehydration followed by centrifugation: (a) RBCs maintain normal volume in isotonic solution; (b) RBCs lost part of their intracellular water in hypertonic solution and became smaller in volume. (c) RBCs were dehydrated to their minimum volume. (d) Excessive dehydration of RBCs. **b)** Osmolality of the supernatants of RBC suspensions after incubation with different saccharides at different concentrations. **c-d)** Pictures and hematocrits of RBC suspensions after incubation with different saccharides at different concentrations and centrifugation. In **Fig. S1c:** The leftmost capillary in each picture is the saline group. From left to right: 0.17 M, 0.33 M, 0.5 M, 0.67 M, 0.83 M, 1.0 M. HCT (%) = ($V_{packed\ RBCs}$ / $V_{RBC\ suspension}$) × 100%.

Tre: trehalose. Suc: sucrose. Mal: maltose. Glu: glucose. Gal: galactose. Fru: fructose. Raf: raffinose.

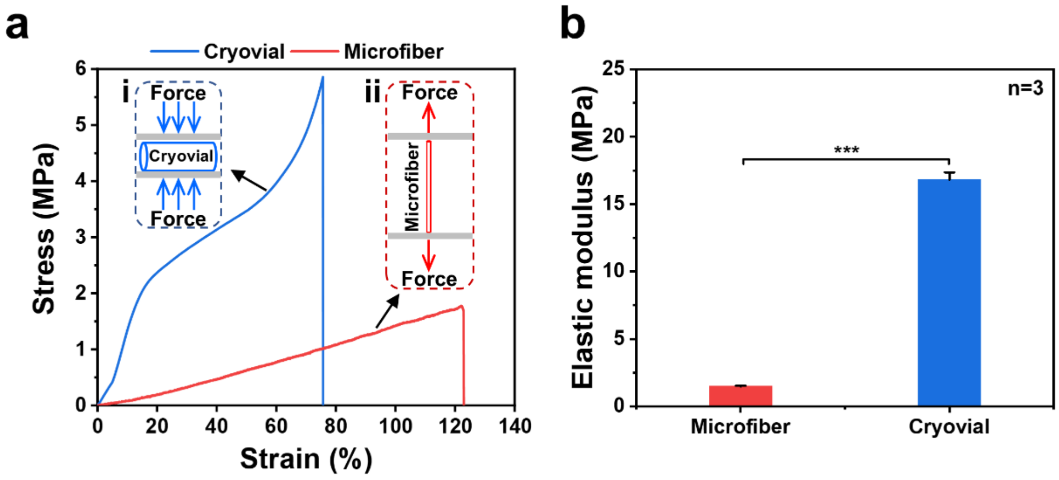

Fig. 2 Mechanical properties analysis at room temperature (25 ℃). **a**) Stress-strain curves of the cryovial and the RBC microfiber. **b**) The elastic modulus of the cryovial and the RBC microfiber

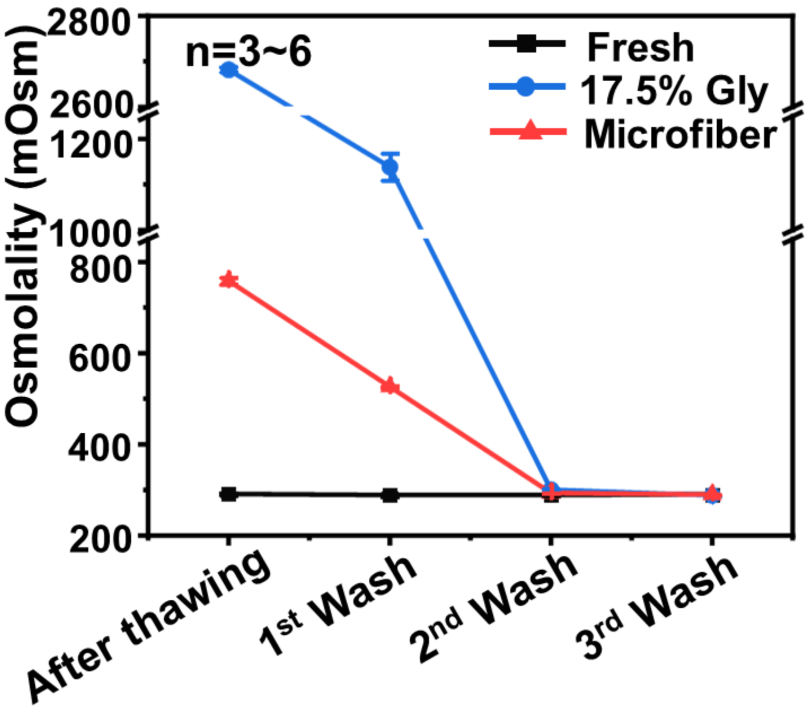

Fig. 3 Osmolality changes at every step during the washing process in the three groups

S1.2 Comparison of the Effects of Trehalose and Other Saccharides on the Cryopreservation of Human RBCs Encapsulated in Microfibers

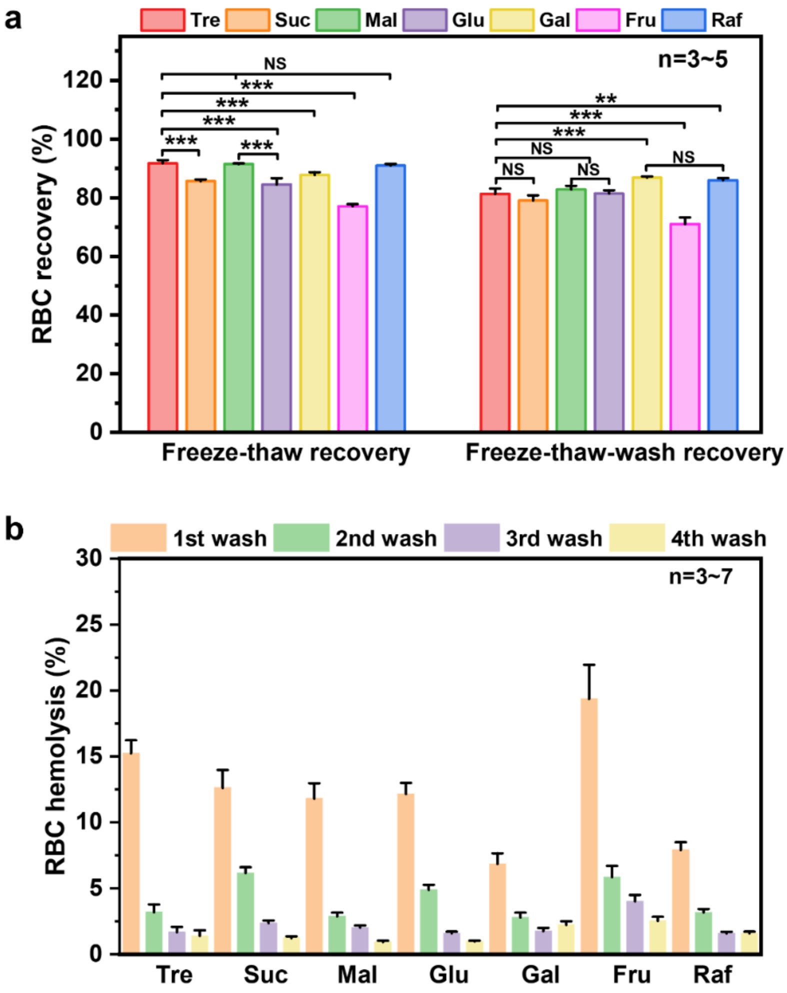

Fig. 4 The cryopreservation of human RBC-laden microfibers with different saccharides at the same concentration of 0.5 M. **a)** The freeze-thaw recovery and freeze-thaw-wash recovery. **b)** The RBC hemolysis at each step during washing

To compare the effects of trehalose and other saccharides on the cryopreservation of human RBCs encapsulated in microfibers, we carried out the cryopreservation experiments using the same procedure and the same concentration of saccharide solutions. As shown in **Fig. S4**, the recovery of RBCs after cryopreservation and washing with different saccharides at the concentration of 0.5 M and hemolysis during washing were studied. Overall, it appeared that the freeze-thaw recovery was higher than 90% in the Tre, Mal, and Raf groups, and there were no significant differences among them (**Fig. S4a**). The freeze-thaw-wash recovery was higher than 80% in all groups except for sucrose and fructose (**Fig. S4a**). The trend of hemolysis in each step of the washing process was similar in all groups. Specifically, there was an obvious hemolysis at the first wash (**Fig. S4b**).

Further, the quality of the washed RBCs was assessed in terms of mechanical property, biochemical property, and morphology. In general, the assessment results were similar for all groups (**Figs. S5 and S6**), except for the ATP levels, which could be recovered in 37 °C human serum and this process occurred on a scale of tens of minutes [S2].

By comparing these data, we could find that for RBCs, lacking the delicate subcellular organelles, predehydration with saccharides such as trehalose at room temperature to minimize osmotic shock and IIF during cooling and the membrabe-stabilization effect of sugars are important during the cryopreservation of human RBCs. More interestingly, glucose, commonly used in clinical practice, also performed well in the cryopreservation of high-hematocrit RBCs encapsulated in microfibers. This will bring great convenience to the clinical application of frozen RBCs.

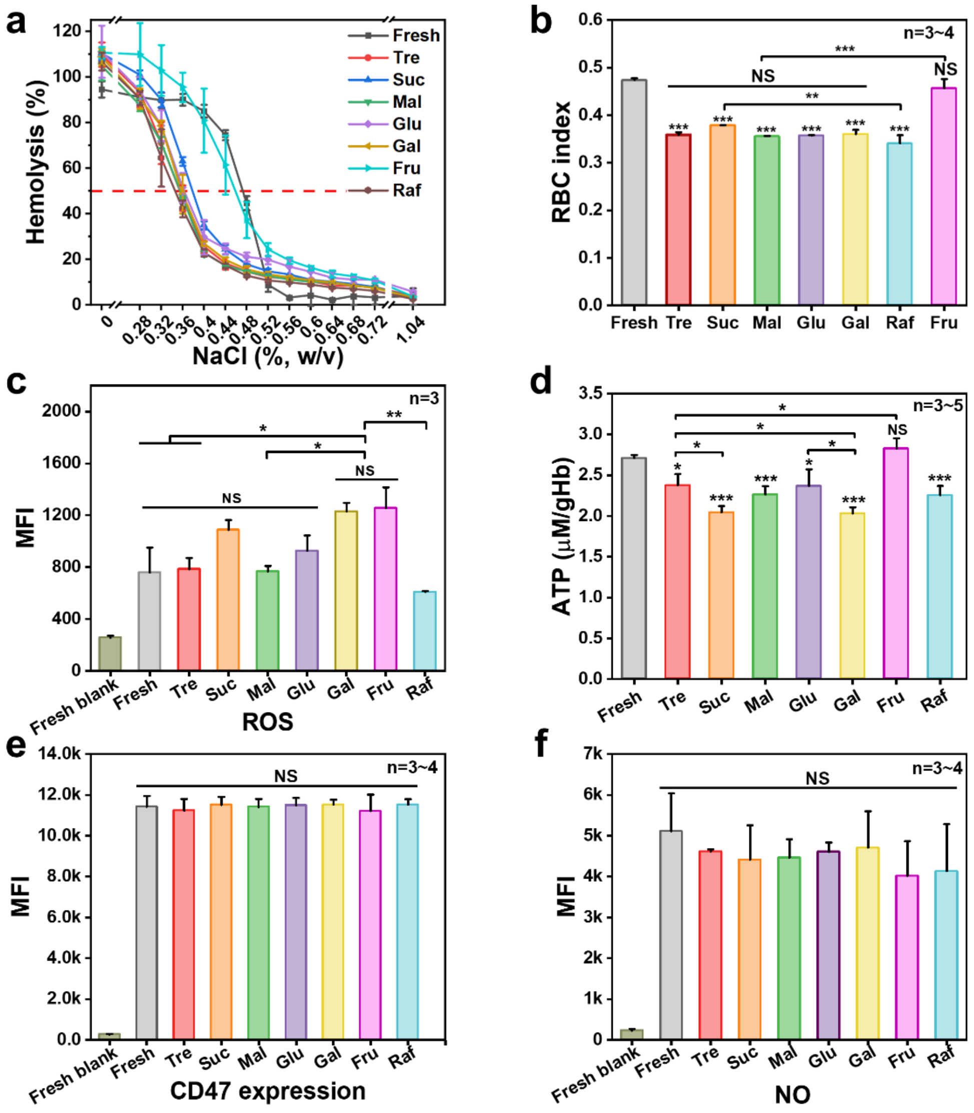

Fig. 5 The assessments of the functional properties of the washed RBCs after cryopreservation with different saccharides. **a)** Osmotic fragility curves of the washed RBCs with different NaCl concentrations. **b)** Osmotic fragility index of the washed RBCs. Three types of characteristic RBC functions were measured: **c)** intracellular

ROS level, **d)** ATP levels, **e)** intracellular CD47 expression level and **f)** intracellular NO level of washed RBCs after cryopreservation

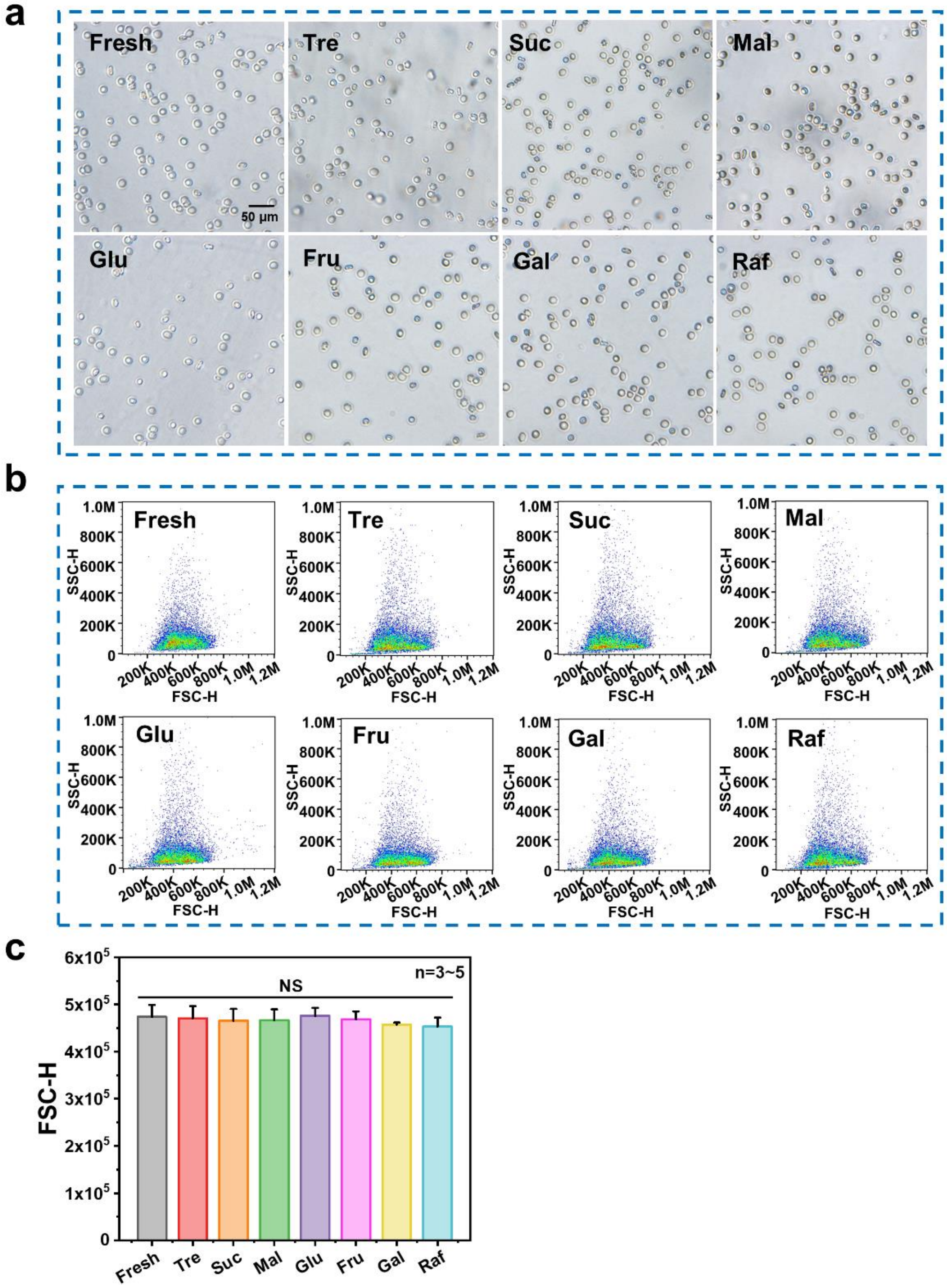

Fig. 6 Assessment of RBC morphology following cryopreservation with different saccharides (0.5 M) and washing process. Micromorphology **(a)** and the forward scatter and side scatter **(b)** of fresh RBCs and washed RBCs after cryopreservation with different saccharides (0.5 M) in the microfiber groups. **c)** The volume change of washed RBCs in different groups

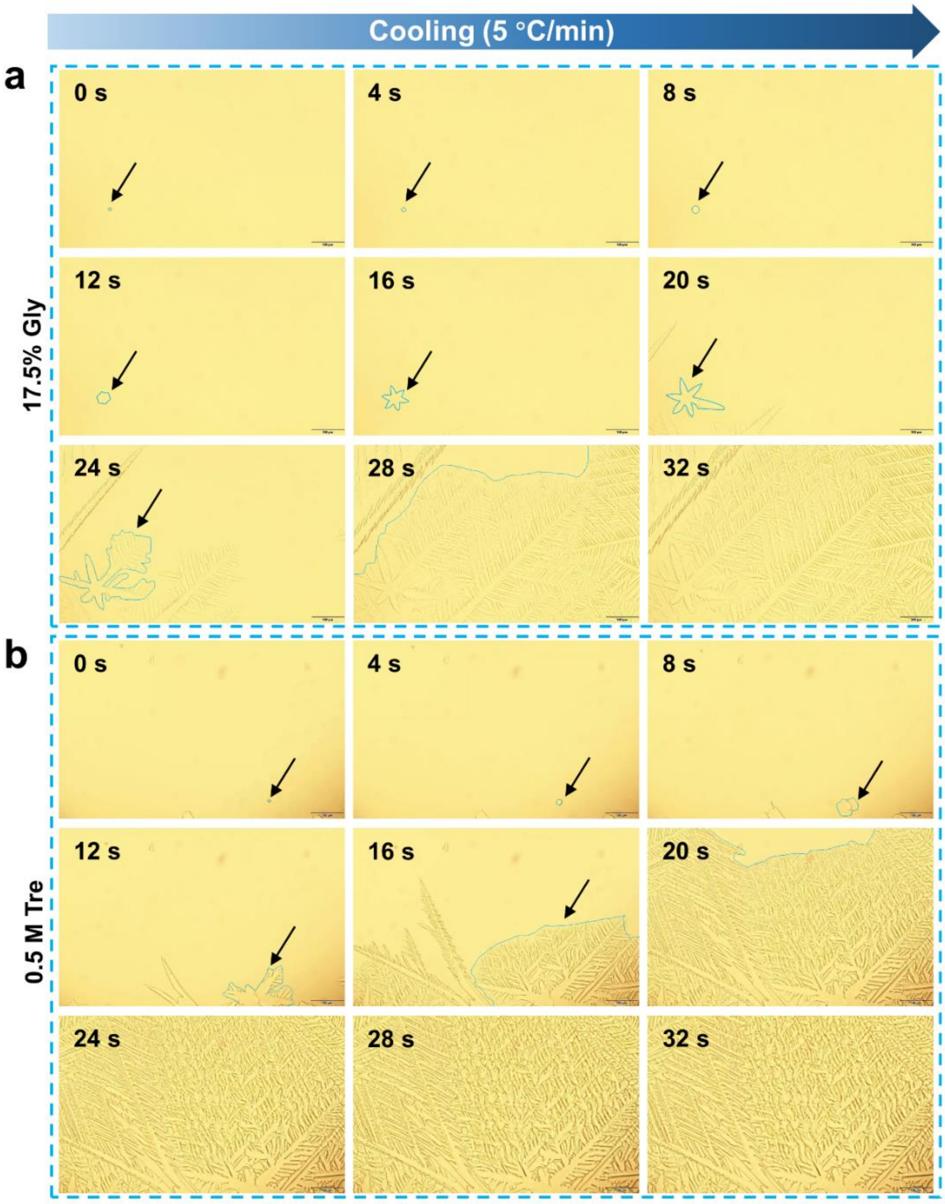

Fig. 7 Photos of ice crystal growth in the 17.5% Gly solution **(a)** and 0.5M Tre solution **(b)** during cooling process

S2 Experimental Methods

S2.1 Materials and Reagents

Sodium alginate (SA), calcium chloride ($CaCl_2$), sodium citrate (SC), trehalose (Tre), NaCl and sucrose (Suc) were purchased from Aladdin Industrial Corporation (Shanghai, China), Hushi Laboratorial Equipment Corporation (Shanghai, China), Biosharp (Hefei, Anhui, China), Sinozyme Biotechnology (Nanning, Guangxi, China), Sangon Biotech (Shanghai, China), and J&K (Beijing, China), respectively. D-fructose (Fru), Maltose (Mal), D-raffinose (Raf) and D-galactose (Gal) were purchased from Macklin (Shanghai, China). Saccharide solutions were used at concentrations of 0.2, 0.4, 0.6,

0.8, 1 and 1.2 M. Sodium alginate was used at 2% (w/v). Sodium citrate was used at 0.3 M. $CaCl_2$ was used at 0.15 M with saccharides of a corresponding concentration.

S2.2 Preparation of RBCs

The human RBC suspensions used in this work were obtained from Hefei Blood Center (Anhui, China). The ethics approval was obtained from the Medical Research Ethics Committee of The First Affiliated Hospital of USTC (ChiCTR1900021038). What's more, the red blood cell suspensions were donated by different healthy volunteers. The RBC suspension was refrigerated at 4 °C for less two weeks. Prior to commencing the experiments, the RBC suspension underwent three rounds of washing with saline solution through centrifugation (using the Centrifuge 5702 R, Eppendorf, US) at 2500 × g for 5 minutes. The supernatant was discarded at each washing. Finally, packed RBCs with a hematocrit (HCT) level of about 80% were obtained. The HCT level was measured through the capillary blood centrifugation (TG12M, Xiangyi, China).

S2.3 Osmolality

The osmolality of the supernatants of RBC suspensions after incubation with different saccharides at different concentrations (0.2, 0.4, 0.6, 0.8, 1.0 and 1.2 M.) was measured by freezing point depression (Osmometer 3250, Advanced Instruments, US).

S2.4 Hematocrit

The hematocrits of RBC suspensions after incubation with different saccharides at different concentrations (0.2, 0.4, 0.6, 0.8, 1.0 and 1.2 M.) were determined by a capillary blood centrifuge (TG12M, Xiangyi, China) and manual calculation. Pictures of RBC suspensions after centrifugation (12000 rpm, 10 min) were taken.

S2.5 Cryopreservation

S2.5.1 Adding CPAs

The packed RBCs and saccharide solutions were mixed into a 5 mL EP tube (BS-15-M, Biosharp, China) at a volume rate of 1:1 to give a hematocrit level of ~40%. The working concentration of the saccharide solutions was 0.5 M. Then, all the samples were kept for 5 min at room temperature for equilibration and pre-dehydration.

S2.5.2 Generation of the RBC-laden Core-shell Alginate Hydrogel Microfibers

2% (w/v) sodium alginate was prepared as the shell solution, and above-mentioned RBC suspension with saccharides was prepared as the inner solution. The fabrication equipment consisted of a tube-in-tube device and two High-Precision Programmable Syringe Pumps (NPZ-010, Nano Apparatus, China). The RBC-laden core-shell alginate hydrogel microfibers were generated from inner solution and shell solution with 0.15 M $CaCl_2$. The flow rate combination 600-200 μL min^{-1} (core-shell) was utilized. Finally, the RBC-laden microfibers were collected in a Petri dish.

S2.5.3 Freezing and Thawing

The surface-dried RBC-laden microfibers were directly immersed in LN_2 for freezing. Then the frozen microfibers loaded with RBCs were thawed in sodium citrate solution pre-warmed at 40 °C placed in a 50 mL centrifuge tube. The hydrogel microfibers were shaken gently and dissolved within half a minute.

S2.5.4 Determination of RBCs Recovery

RBC recovery was determined by evaluating the amount of hemoglobin in the supernatants of the samples. RBCs suspension was centrifuged at 2500 × g for 5 minutes after thawing to collect the supernatants. Then, the absorbance of the supernatant at 415 nm was measured by using a microplate photometer (Thermo Multiskan FC, Thermo Scientific, US). Thus the hemolysis and recovery of RBCs were calculated according to the following formula [S3]:

$$\text{RBC Recovery } (\%) = 100\ \% - \left(\frac{A - A_0}{A_1 - A_0}\right) * 100\ \%$$

Where A, A_0, and A_1 are the absorbance of the supernatant of the sample, the positive control group, and the negative control group, respectively.

S2.5.5 Washing

After thawing, all the samples were centrifugated at 2500 × g for 5 minutes, and then the supernatant was removed. Furthermore, the packed RBCs were pipetted into a 15 mL centrifuge tube. Subsequently, the microfiber group chose0.9% NaCl solution as the washing solution in the three washing steps. The absorbance of the supernatant was measured after every washing step to evaluate hemolysis.

S2.6 Evaluations of RBCs after Cryopreservation

S2.6.1 Osmotic Fragility

The osmotic fragility of the RBCs was measured by using dilutions of 1.04% NaCl solution. The 1.04% NaCl solution was diluted with water to 0.28%-0.72% NaCl solutions in increments of 0.04%. The packed washed RBCs after cryopreservation were diluted 1:100 in various dilution buffers and equilibrated for 2 h at room temperature. Afterward, the samples were centrifuged to measure the absorbance of the supernatant as above. The osmotic fragility index was calculated by the concentration of NaCl solution inducing 50% hemolysis.

S2.6.2 Biochemical Property

After cryopreservation and washing, the biochemical properties of RBCs were determined, including intracellular ROS levels, ATP, intracellular NO levels, and CD47 expression. The relevant assay kits operated in light of the suppliers' instructions were the same as those shown in our previous publications [S4].

S2.6.3 Morphological Evaluation

The micrographs of RBCs after cryopreservation and washing were acquired by a microscope (BX51, Olympus, Japan). What's more, flow cytometry (CytoFLEX, Beckman) was also used to test and analyse the morphology of RBCs.

S2.7 Analysis of Ice Crystal Growth Rate of CPAs

The cryomicroscope was used to observe the ice crystal growth during cooling process. First, an extremely small amount of SiO_2 microspheres was added to the CPAs. After mixing, 3 μL of the mixture was taken and dropped over the coverslips which had been cleaned with alcohol. The sample was placed on the cryostage and cooled at a cooling rate of 20 °C /min until completely frozen. Then, the sample was rewarmed to a temperature slightly lower than its melting temperature, leaving only a small ice nucleus remaining in the field of view. After that, the ice nucleus was cooled down to -100 ℃ at 5 ℃/min. A camera was used to record the entire cooling process.

S2.8 Mechanical Performance Tests

S2.8.1 Compression test

The tail and cover of the cryovial were removed and placed on the center platform of the tensile machine (Reger RGM10, Shenzhen Reger Instrument Co., Ltd., China). The test distance was adjusted by gradually lowering the upper platform until it was on the verge of contacting the sample's surface. Subsequently, the Reger software was opened, and the force and displacement output signals were reset. The compression experiment was initiated and continued until the cryovial was compressed and fractured.

S2.8.2 Tensile Test

RBC microfibers, each measuring 2-3 cm in length, were prepared and affixed to the tensile machine (Dongguan Wisdom Precision Instrument Co., Ltd., China) using a fixture. The fixture was then adjusted to maintain the fibers in a naturally vertical position. The testing software was launched to configure parameters, reset force and displacement output signals, and subsequently initiate the stretching process until the fibers fractured.

Movie S1 Freezing of the 17.5% Gly group

Movie S2 Thawing of the 17.5% Gly group

Movie S3 Freezing of the Microfiber group

Movie S4 Thawing of the Microfiber group

Movie S5 Cryomicroscopic study of Gly solution

Movie S6 Cryomicroscopic study of Tre solution

Supplementary References

[S1] H. Huang, G. Zhao, Y. Zhang, J. Xu, T.L. Toth, X. He, Predehydration and Ice Seeding in the Presence of Trehalose Enable Cell Cryopreservation. ACS

Biomater. Sci. Eng. **3,** 1758–1768 (2017). https://doi.org/10.1021/acsbiomaterials.7b00201

[S2] Z. Xu, W. Dou, C. Wang, Y. Sun, Stiffness and ATP recovery of stored red blood cells in serum. Microsyst Nanoeng. **5**, 1–9 (2019). https://doi.org/10.1038/s41378-019-0097-7

[S3] V. Han, K. Serrano, D.V. Devine, A comparative study of common techniques used to measure haemolysis in stored red cell concentrates. Vox Sang. **98**, 116–123(2010). https://doi.org/10.1111/j.1423-0410.2009.01249.x

[S4] L. Shen, X. Qin, M. Wang, D. Gao, X. Ouyang, G. Zhao, Combining Cooling Enhancement and Trehalose Dehydration to Enable Scalable Volume Cryopreservation of Red Blood Cells with Low Concentration of Glycerol. Adv Eng Mater. 2200835 (2022). https://doi.org/10.1002/adem.202200835
